# Assessing the potential for deep learning and computer vision to identify bumble bee species from images

**DOI:** 10.1038/s41598-021-87210-1

**Published:** 2021-04-07

**Authors:** Brian J. Spiesman, Claudio Gratton, Richard G. Hatfield, William H. Hsu, Sarina Jepsen, Brian McCornack, Krushi Patel, Guanghui Wang

**Affiliations:** 1 Department of Entomology, Kansas State University, Manhattan, KS USA; 2 Department of Entomology, University of Wiscosin – Madison, Madison, WI USA; 3 The Xerces Society for Invertebrate Conservation, Portland, OR USA; 4 Department of Computer Science, Kansas State University, Manhattan, KS USA; 5 Department of Electrical Engineering and Computer Science, University of Kansas, Lawrence, KS USA; 6 Department of Computer Science, Ryerson University, Toronto, ON Canada

**Keywords:** Machine learning, Ecology, Biodiversity, Community ecology, Conservation biology

## Abstract

Pollinators are undergoing a global decline. Although vital to pollinator conservation and ecological research, species-level identification is expensive, time consuming, and requires specialized taxonomic training. However, deep learning and computer vision are providing ways to open this methodological bottleneck through automated identification from images. Focusing on bumble bees, we compare four convolutional neural network classification models to evaluate prediction speed, accuracy, and the potential of this technology for automated bee identification. We gathered over 89,000 images of bumble bees, representing 36 species in North America, to train the ResNet, Wide ResNet, InceptionV3, and MnasNet models. Among these models, InceptionV3 presented a good balance of accuracy (91.6%) and average speed (3.34 ms). Species-level error rates were generally smaller for species represented by more training images. However, error rates also depended on the level of morphological variability among individuals within a species and similarity to other species. Continued development of this technology for automatic species identification and monitoring has the potential to be transformative for the fields of ecology and conservation. To this end, we present BeeMachine, a web application that allows anyone to use our classification model to identify bumble bees in their own images.

## Introduction

Bees (Hymenoptera: Anthophila) serve a critical role in most terrestrial ecosystems as pollinators of crops and natural plant communities e.g.,^1–3^. With mounting evidence for the global decline of many bee species^4–7^, considerable effort has been focused on understanding the causes of bee decline, monitoring programs, and developing conservation strategies to preserve their biodiversity and ensure the continued provision of pollination services^8^. One essential yet challenging step in bee research is to accurately identify individuals so that the number of species and population sizes in an area can be assessed. Focusing on bumble bees (*Bombus*) in the United States and Canada, we address this challenge in bee research by assessing the potential for automatic species-level identification from images using deep learning classification models.

Species-level bee identification is challenging because experts often rely on subtle morphological features to differentiate many of the more than 20,000 species worldwide^9^. Bumble bees, for example, can often be identified by experts in the field or from images based on distinctive color patterns. However, similarities between some species require comparing very fine-scale differences in e.g., coloration, facial features, or genitalia^10,11^. As a result, many species cannot be identified in the field or from photos by human observers if important features are obscured or not sufficiently resolved. Instead, individuals are usually collected, cleaned, pinned, and labeled, before being identified by experts under a microscope. This process is expensive, time consuming, and greatly slows the pace of pollinator research. The challenge will only become more limiting with the declining number of taxonomic experts^12^. The remaining experts are underfunded and have limited time, which can be consumed by identifying numerous samples of common species^13^. Moreover, this sampling process requires lethal collection methods, which are increasingly disparaged or restricted, especially when projects involve sensitive species, such as the endangered rusty patched bumble bee (*Bombus affinis*). Methods for automated and reliable bee identification from photos are thus greatly needed^14^.

In addition to the benefits for basic research, tools for automated identification would also benefit community (or citizen) science programs, which provide important monitoring data and engage the public in science and conservation efforts. With programs such as BeeSpotter (beespotter.org), Bumble Bee Watch (BBW; bumblebeewatch.org), iNaturalist (inaturalist.org), and the Wisconsin Bumble Bee Brigade (WBBB; wiatri.net/inventory/bbb), users can contribute to national and regional databases by uploading georeferenced images of bumble bees and providing preliminary identifications, which are then verified by experts or community sourced. This verification step is important because user-submitted identifications to WBBB and BBW agree with experts only 73 and 53 percent of the time, respectively^15,16^. If not properly verified, such erroneous data could have serious negative consequences for pollinator conservation. Moreover, automated identification methods would reduce the substantial number of submissions that remain unverified because experts cannot keep up, while providing high quality data for pollinator conservation science.

There has been a longstanding recognition that we need tools for automated insect identification^17–19^, however we have been limited by effective methods and computational power. But, with the use of powerful graphics processing units and ongoing advances in computer vision, it is now possible to efficiently and accurately detect and identify objects, including insects, from images. Applying state-of-the-art deep learning methods to the problem of species-level bee identification would help reduce this research bottleneck and put an expert-level identification tool in the hands of everyone from bee enthusiasts to students, educators, land managers, and scientists^14^. Deep learning technology has a realistic potential to be transformative, not only for pollinator research, but for addressing a wide range of problems in agriculture^20^ and surveillance of arthropods that transmit human pathogens^21^.

Deep learning techniques such as convolutional neural networks (CNNs) are at the forefront of computer vision. More commonly applied in the fields of self-driving cars^22^ and healthcare diagnostics^23^, researchers are beginning to apply CNNs to insect detection and identification^21,24–26^. Mobile apps such as Seek (inaturalist.org/pages/seek_app) and Google Lens (lens.google.com) can be used to identify some taxa but species-level accuracy for bees in these apps is not sufficient for research purposes.

One key benefit of CNNs that makes them ideal for bee identification is that they do not rely on inputs of known feature sets, such as the morphological characteristics that taxonomists rely on. Instead, training a CNN only requires a set of labeled images that the model can learn from, developing its own feature set that it uses for identification. CNNs are robust to images with subjects (e.g., bees) that are oriented differently, partially obscured, or set in different environmental contexts, such as in different lighting conditions or visiting different flower species. This ability to self-learn discriminating feature sets is ideal for a species-level bee classifier because key features that experts require to make identifications are often not visible in images.

In this paper we assess the performance of four convolutional neural network models trained to classify 36 North American bumble bee species, comparing tradeoffs between speed and accuracy. We then assess the potential for deep learning to automate the identification of bumble bee species from images and discuss the outlook for applying this technology in ecological research and large scale monitoring programs. Lastly, we introduce BeeMachine, a web app based on our model that allows users to identify bumble bees from their own images.

## Methods

### Image data set

We focused our analysis on bumble bees in the United States and Canada, as described in Williams et al.^27^. To train and validate classification models, bumble bee images were gathered from Bumble Bee Watch, iNaturalist, and BugGuide. Only images categorized as "verified" (identified by an expert) or "research grade" (identity agreed upon by at least two of three users) from Bumble Bee Watch and iNaturalist, respectively, were included in our analyses. Images from BugGuide were identified by expert naturalists. Our initial dataset comprised over 120,000 images belonging to 42 species.

**Figure 1:**
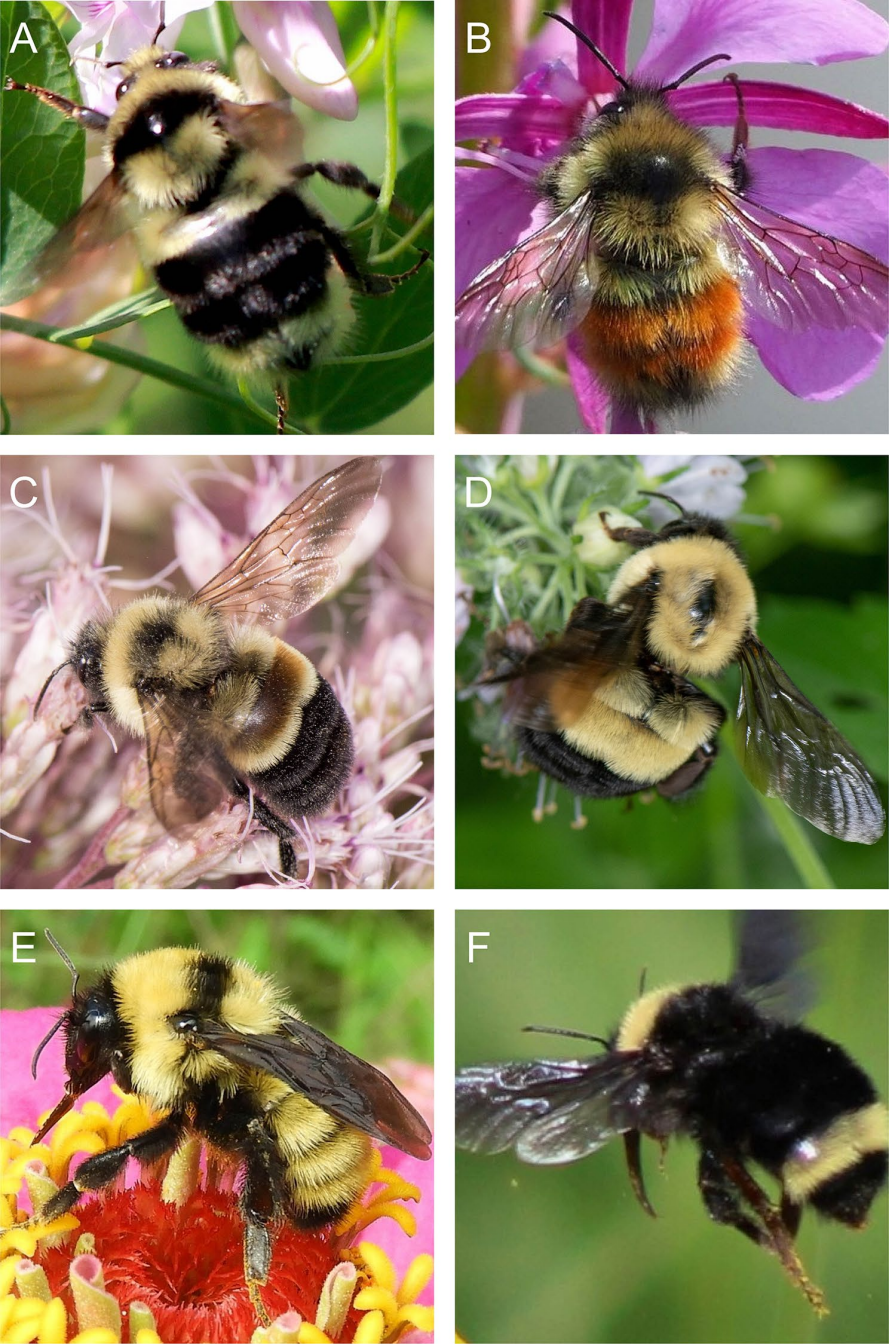
Example test images showing coarse scale intraspecific variability in appearance. *Bombus melanopygus* are (**A**) without orange coloration in the southern part of their range but (**B**) with orange on the northern end. (**C**) A *B*. *affinis* worker displaying its characteristic rusty-colored patch on T2 of its abdomen, however (**D**) the patch is usually absent or reduced on queens. *B*. *fervidus* has (**E**) extensive yellow coloration in the eastern part of its range, but (**F**) is more extensively black in the western end. Photo credits: (**A**) Andrea Kreuzhage, (**B**) Scott Ramos, (**C**, **D**) Heather Holm, (**E**) Sue Gregoire, (**F**) Asa B. Spade.

Original images were cropped tightly to each bee using an object detection algorithm trained to detect bumble bees (Fig. 1). This allowed us to automate some of the preprocessing of the image data set. Each cropped image was encoded to the JPG format and then manually inspected for errors. Detection algorithm errors included false positives, such as other bee taxa that happened to be included in an image, which were discarded. Rarely, misidentified bumble bees were encountered, which we relabeled and included in our training set. Cropped images less than 200 × 200 pixels were discarded. We did not distinguish between workers, queens, and males within species so our classification models generalize across bumble bee castes that often vary in morphology and color pattern.

**Figure 2:**
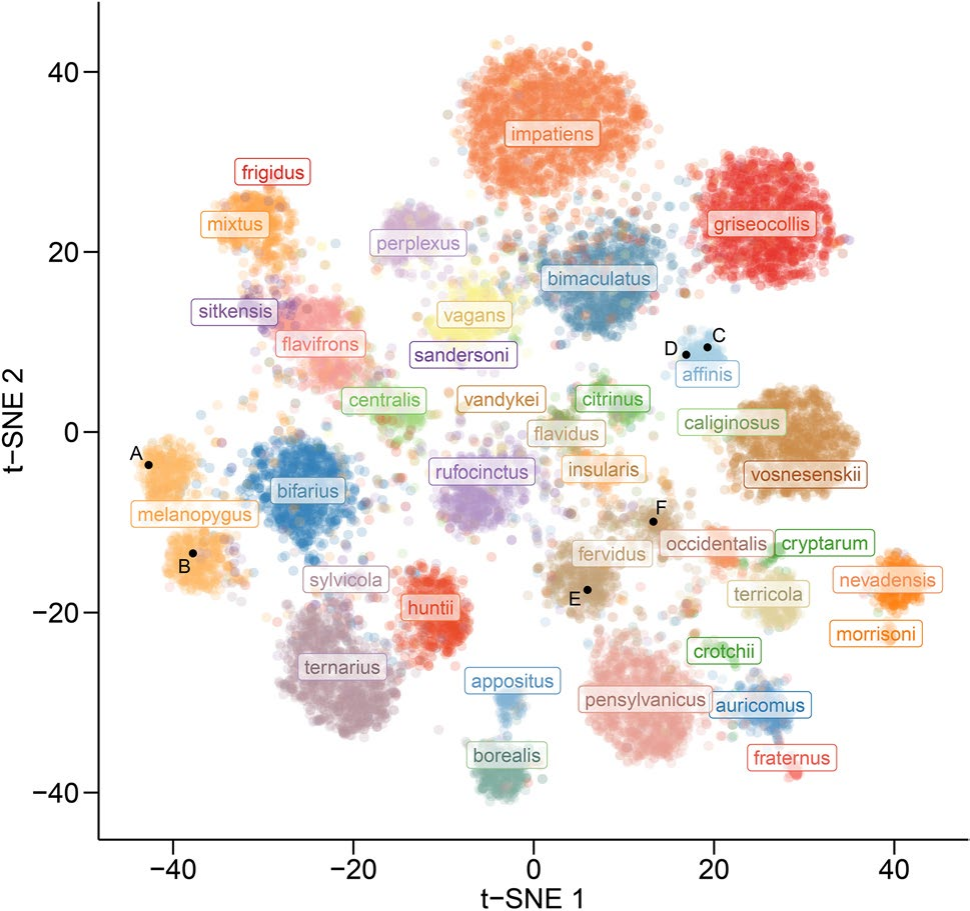
Visualization of trait separation among species based on t-SNE. Black points (**A–F**) show the location in trait space of images in Fig. 1 panels.

We required a minimum of 150 images for a species to be included in the analysis. The sample sizes of 6 species in our original dataset were thus insufficient to be included in the model. After quality control we retained 89,776 images belonging to 36 species (Fig. 2, Table S1) out of the 46 bumble bee species in the US and Canada recognized by Williams et al.^27^. Some species are more frequently photographed than others because they are common and/or live near population centers, while others are rarely photographed. Six of the North American species not included (*B*. *balteus*, *B*. *distinguendus*, *B*. *hyperboreus*, *B*. *jonellus*, *B*. *neoboreus*, and *B*. *polaris*) are high-latitude and/or high-elevation species that are infrequently encountered. Three species (*B*. *bohemicus*, *B*. *suckleyi*, and *B*. *variabilis*) are historically uncommon and/or in decline. One species, *B*. *franklini*, has a very restricted range and has not been encountered since 2006 despite a concerted search. As a result of this range in commonness, our image dataset was highly imbalanced among species. We therefore limited the number of images per species to a maximum of 10,000 to help limit the classification bias associated with imbalanced data sets.

### Classification models

Many classification models based on convolutional neural networks (CNN) have been proposed in the field of computer vision. We compared the performance of four widely used models that vary in complexity of architecture to evaluate tradeoffs in accuracy and speed for bumble bee species classification. One of the main limitations of deep neural networks is the vanishing gradient problem, which prevents the adjustment of model weights during the training process, making it hard to improve model performance before information passes through the end of the network. (1) ResNet-101^28^ was proposed to address this problem by using skip connections that allow useful layer output to pass over groups of layers (or residual blocks) and thus penetrate much further into deep networks. (2) Wide-ResNet-101^29^, on the other hand, is wider but shallower than ResNet-101. This difference in architecture allows information to more easily pass through the network while providing a greater number of channels to maintain performance. (3) InceptionV3^30^ has fewer layers but achieves greater computational efficiency by, for example, using factorized convolution, which breaks down channels into simpler linear sequences while maintaining spatial context within the image. (4) MnasNet-A1^31^ was designed to be faster for mobile devices and thus has the fewest layers of the models compared here. The MnasNet architecture was obtained by a mobile neural architecture search mechanism that is designed to find an optimal trade-off between accuracy and latency.

### Model training

To speed up the training process, we used models pre-trained on the ImagNet database^32^ and then retrained them for bumble bee species classification using our image data set. We divided our full data set into training (80%) and test (20%) groups. After randomly shuffling images in each class (species) we split the images within species along the 80:20 ratio to maintain their proportional representations in the training and test groups. For each model, cropped images were resampled to a standard size for model input. Following the original implementation of these models, we used images of 224 × 224 pixels for ResNet, Wide-Resnet, and MnasNet, and 299 × 299 pixels for InceptionV3. We used an image augmentation strategy that included random rotation (≤ 100°), crop (≤ 10%), sheer (≤ 30%) and horizontal flip to help reduce overfitting and improve the generality of our models^33^. To help account for the class imbalance in our data set, predictions were weighted by class sample size. We used the SGD (stochastic gradient descent) optimizer with an initial learning rate of 0.01 for all models except MansNet, which was initially set at 0.1. The learning rate was reduced by a factor of 10 after steps of 30 epochs. We used batch normalization and models were trained for 150 epochs using Nvidia Tesla K80 or P100 GPUs.

At the species level, we calculated two metrics of model performance: precision (true positives / (true positives + false positives)) and recall (or sensitivity: true positives / (true positives + false negatives)). Precision is a useful metric when using the model to predict the identity of an unknown specimen. That is, given a prediction, precision tells you how likely it is that the prediction is correct. Recall, on the other hand, lets a user assess, given a specimen with a known label or identification, how likely it is that the model will make the correct prediction. Species-specific error rates were defined as 1 – precision or the false positive rate.

For each model, we compared overall (top-1) accuracy, or the accuracy of the most likely prediction. We compared top-N test accuracy (i.e., accuracy assuming the correct identity is within the top-N predictions), macro precision (i.e., the mean of species-level recall scores), and macro recall (i.e., mean of species-level precision scores). We also examined the tradeoff in overall test accuracy and speed to determine the most appropriate model to focus on for this study. Model speed was quantified as the mean time necessary to make predictions on images in the test data set. All speed tests were run on the same system using a Tesla P100 GPU. We then examined species-level precision, recall and error rates in relation to the number of training and test images. We visualized model-based trait separation among species using T-distributed Stochastic Neighbor Embedding (t-SNE) by examining model weights from the final fully connected layer of the network before softmax predictions were made.

## Results

Three of the CNN models used for species-level identification of bumble bees all provided similar accuracy rates (Table 1). Wide-ResNet had the highest test accuracy of 91.7% followed closely by InceptionV3 (91.6%) and ResNet100 (91.3%). MnasNet, however, had relatively low test accuracy of 85.8%. There was a substantial increase of 5.4 percentage points on average, between top-1 and top-2 accuracy. Top-5 accuracy was greater than 98.1% for all models. There was a tradeoff between speed and accuracy for the best and fastest models with Wide-ResNet being slightly more accurate but relatively slow and MnasNet being slightly faster but relatively inaccurate.

**Table 1:** Comparison of model size, speed, and performance, ordered by top-1 accuracy. Text in bold indicates the best value in each category. Wide-ResNet101 has the highest top-1 accuracy and macro precision but was substantially slower than the other models. MnasNet-A1 was the fastest model but had relatively poor performance. InceptionV3 was relatively fast while maintaining good model performance with the highest precision and second highest accuracy and recall.

Model	#Params (million)	Model speed (ms)	Top-1 accuracy	Top-2 accuracy	Top-3 accuracy	Top-4 accuracy	Top-5 accuracy	Macro recall	Macro precision
Wide-ResNet101	124.9	5.46	**0.9171**	0.9627	0.9782	0.9850	**0.9897**	**0.8552**	0.8831
InceptionV3	24.0	3.34	0.9162	0.9610	0.9767	0.9834	0.9882	0.8519	**0.8881**
ResNet101	42.6	3.33	0.9133	**0.9633**	**0.9787**	**0.9852**	0.9892	0.8499	0.8740
MnasNet-A1	**1.0**	**3.28**	0.8579	0.9335	0.9609	0.9730	0.9814	0.7689	0.8250

Of the four models tested, InceptionV3 presents a good balance between performance and speed. InceptionV3 had the highest precision and nearly matched Wide-ResNet’s accuracy and recall while being 39% (2.1 ms) faster (Table 1). Likewise, InceptionV3 was 5.8 percentage points more accurate than MnasNet while only 0.06 ms slower. The speed and accuracy of InceptionV3 makes it useful for web-based and mobile applications that rely on both speed and reliable predictions. We therefore focus on our InceptionV3 results for the remainder of this paper.

The InceptionV3 model nicely separated species into distinct groupings based on traits extracted from the model (Fig. 2). There was little overlap in two-dimensional trait space, which corresponds with the high degree of accuracy in the classification results.

The classification results for each test image are displayed in a confusion matrix (Table. 2, see also Table S2), which shows how predicted species (columns) correspond to the actual species (rows). Values along the diagonal indicate the number of correct predictions, whereas off-diagonal values indicate misclassifications. *Bombus affinis*, for example, was correctly classified, based on the match to taxonomic expert classification, in 250 of 258 test images (96.9%) indicating high recall. Similarly, only *B*. *borealis*, *B*. *fervidus*, *B*. *perplexus*, and *B*. *rufocinctus* were mistaken for *B*. *affinis* in 1, 1, 1 and 3 of their respective test images, indicating that *B*. *affinis* has high precision (97.7%).

**Table 2:**
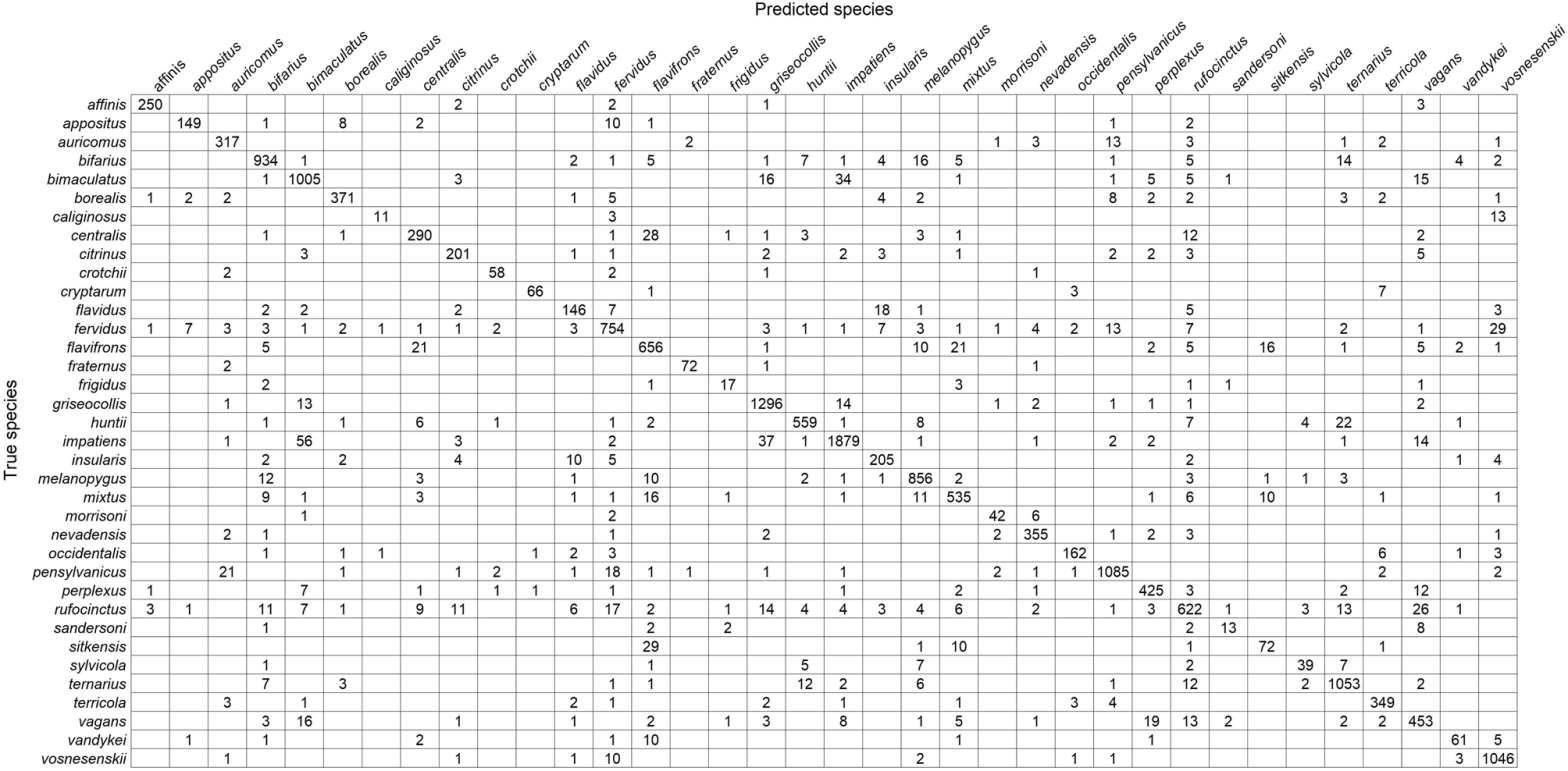
InceptionV3 confusion matrix showing the number of test images for each prediction.

*Bombus affinis* had high recall and precision, with few instances of other species being mistaken for *B. affinis* and mistaking *B. affinis* for few other species. *Bombus vosnesenskii*, *B. griseocollis*, *B. fraternus*, *B. pensylvanicus*, and *B. impatiens* also performed well on both metrics. On the other hand, *B*. *sandersoni*, *B. caliginosus*, *B*. *sitkensis*, *B*. *sylvicola*, and *B*. *frigidus* performed the most poorly on these two metrics. *Bombus caliginosus* is very similar in appearance to *B*. *vosnesenskii* (Fig. 2) and was therefore frequently misclassified as such (Table 2). Likewise, *B*. *sandersoni* is similar in appearance to *B*. *vagans* (Fig. 2), with which it was most often confused (Table 2).

**Figure 3:**
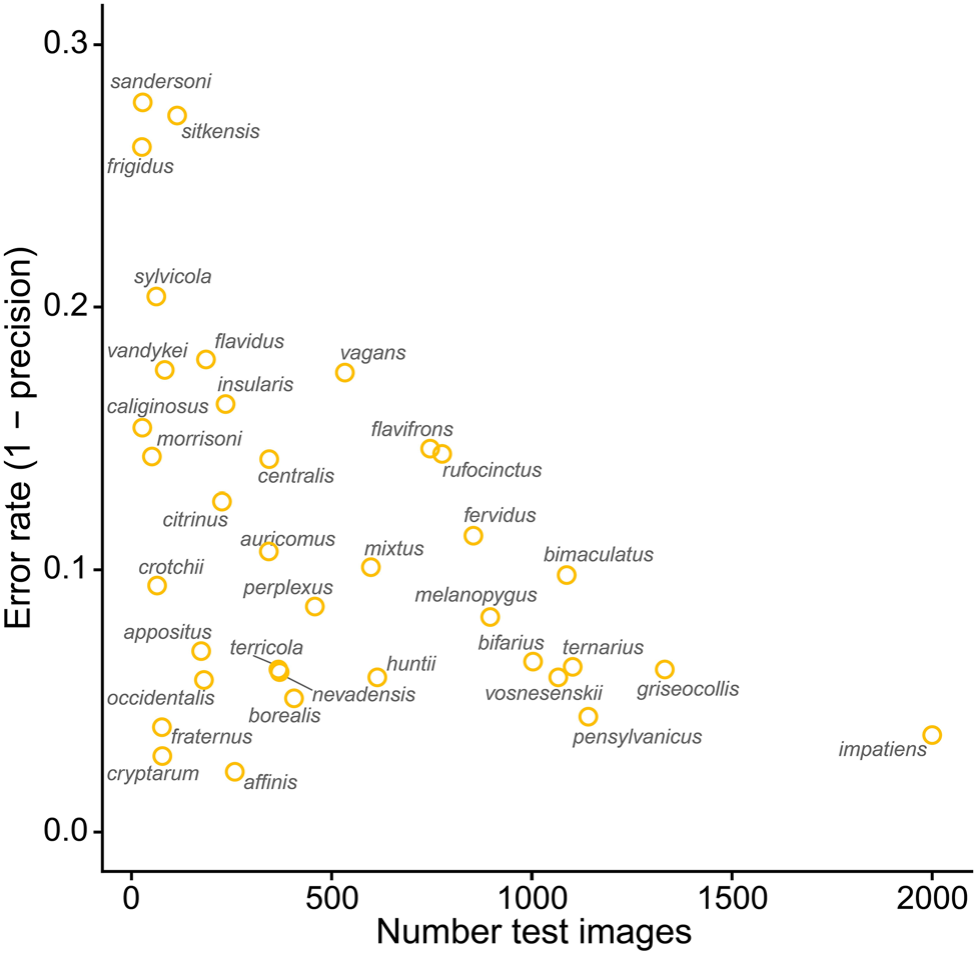
Relationship between error rate and the number of test images.

**Figure 4:**
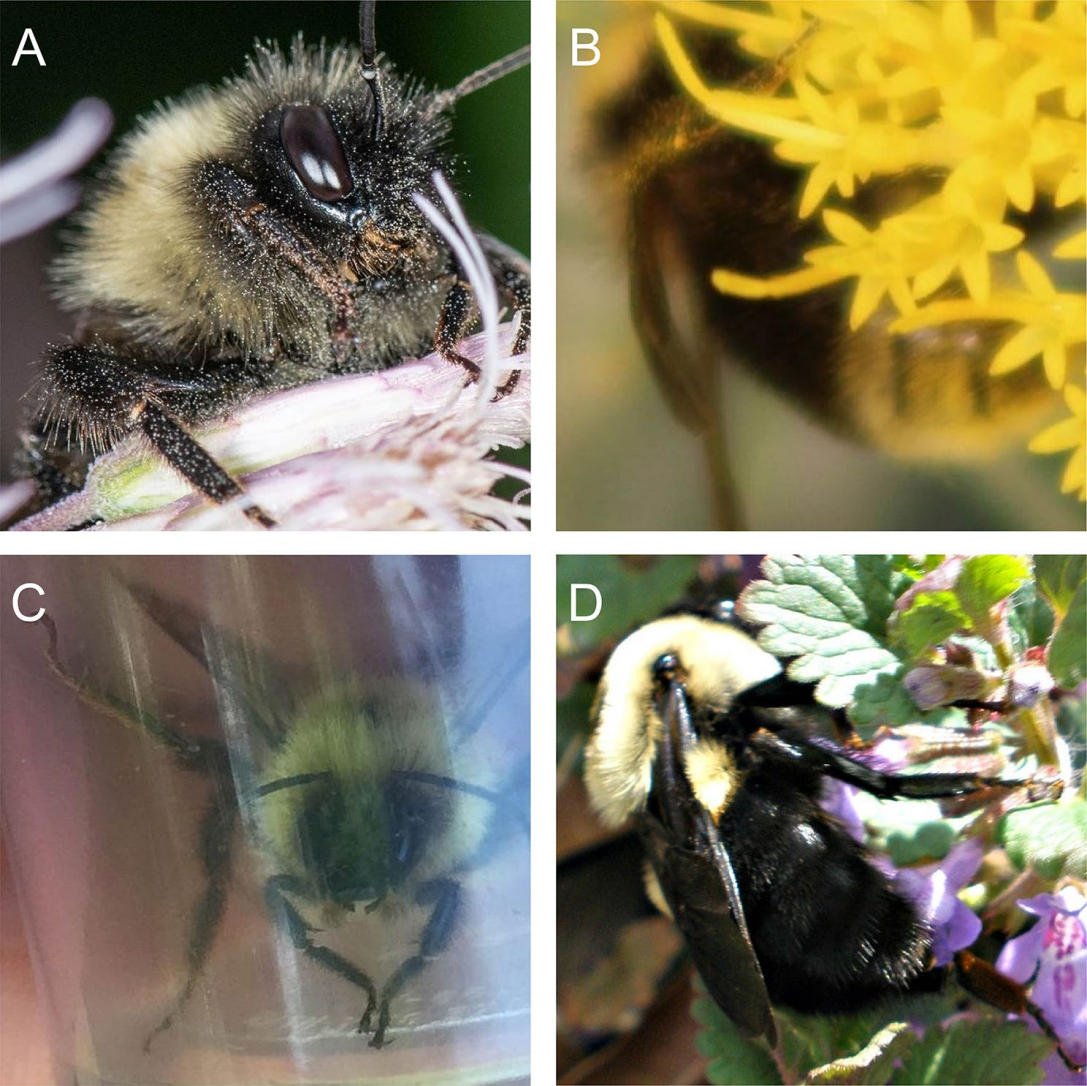
Examples of misclassified images. (**A**) *B*. *affinis* misclassified as *B*. *citrinus*. (**B**) *B. occidentalis* misclassified as *B. terricola*. (**C**) *B. bimaculatus* misclassified as *B. impatiens*. (**D**) *B. griseocollis* misclassified as *B. impatiens*. Photo credits: (**A**) Heather Holm, (**B**) Bernard Yurke, (**C**) Sarah Litterick, (**D**) Ansel Oommen.

Species that were trained on more images tended to have lower test error rates (Fig. 3). However, there was substantial variation in error rates for species with low sample sizes, which was likely due to the degree of intraspecific variation and/or distinctiveness from other species. Regardless of species, images were more likely to be misclassified if bees were in poor focus or obscured. Examples of misclassified images are shown in Fig. 4.

To assess how model results were affected by the particular random subsets of training and test images, we trained each model two additional times with different random train/test splits. These subsequent model runs provided very similar results compared to those reported above, with on average less than one percentage point difference in top-1 accuracy, macro precision, and macro recall.

## Discussion

Using computer vision to identify bees or other taxa in images will be important for advancing research on pollinator ecology and conservation. We have shown that a deep learning image classification approach can accurately (> 91% for our best models) and rapidly identify North American bumble bee species from heterogeneous digital images. It is not known what the true range of expert accuracy is, but as with our model, it likely varies by species and with the quality of images available to make a prediction. Anecdotally, our model correctly classified a test image that we later discovered was mislabeled by an expert; a small degree of mislabeling is unavoidable in large image datasets. This observation is consistent with research showing that CNN models are robust to a small level of mislabeling^34^. It also suggests that with refinement, deep CNN models will have the potential to perform at least on par with experts at image classification tasks.

The four CNN models we tested performed similarly. However, InceptionV3, with its relatively small size and complex architecture, presented a good blend of speed and accuracy. Although smaller models, such as MnasNet-A1, can be slightly faster, they can suffer from lower accuracy, recall, and precision, compared to InceptionV3. Potential end users of this classification model, i.e., those interested in characterizing bee communities and monitoring population trends, would likely favor reliability over model speed and latency issues. However, speed is a factor in user satisfaction and will be important for future video-based applications of our model. Nevertheless, minimizing classification error is especially important when using crowdsourced data with inexperienced observers to monitor endangered species. For example, using error-prone data can over- or underestimate spatial distributions depending on whether misclassifications are false positives or false negatives^35,36^, which can significantly affect conservation management decisions.

At the species level, InceptionV3 classification error rates decreased with a greater number of training and test samples. But error rates also depended on a species’ degree of variability in appearance. For example, *B. fraternus* is easily identified by experts because of its relatively distinctive appearance that varies little among castes or across its North American range. Thus, *B. fraternus* had a relatively low error rate (4.0%) despite being trained on few images (n = 304). On the other hand, *Bombus sylvicola* has a similar training sample size (n = 248) but a much higher error rate (20.4%). This is likely because *B. sylvicola* is more variable in appearance and can resemble many other species, such as *B*. *huntii*, *B*. *melanopygus*, *B*. *rufocinctus*, and *B*. *ternarius*. Similarly, *Bombus rufocinctus* is highly variable in appearance across its broad North American range^27^ and was confused with 25 other species, the most in our study. However, the larger number of training images of *B. rufocinctus* (n = 3,104) appeared to help reduce its error rate (14.4%). *Bombus caliginosus* also had a small training sample size (n = 108) and relatively high error rate (15.4%). But instead of having a variable appearance, it is remarkably similar to *B*. *vosnesenskii*, with which it was frequently confused in our model. Indeed, experts can struggle to differentiate these two species based on photos alone.

Even species with many samples may sometimes be confused within a smaller group of similar species with similar morphology. For example, *Bombus bimaculatus*, *B*. *impatiens*, and *B*. *griseocollis* share similar features and are thus grouped together in feature space (Fig. 2). Figure 4D shows an example of *B*. *griseocollis* that was misclassified as *B*. *impatiens*. *Bombus griseocollis*, however, was the second most likely choice. *Bombus pensylvanicus* and *B*. *auricomus* are similarly grouped in feature space. There can be a relatively small difference in prediction probability scores among species in these small groups of similar yet highly sampled species, which is one of the reasons for the large 5.4 percentage point increase from top-1 to top-2 model accuracy. That is, if the top prediction is not correct, the top two predictions are very likely to include another species within the multispecies grouping and thus contain the correct class.

Gathering more images, especially of species with higher error rates, would likely improve the classification accuracy of our model and reduce species-specific error rates. This would allow us to capture a greater range of the heterogeneity in each species as well as reduce the imbalance among classes. For example, species with at least 4,500 images (3,600 train + 900 test) all had error rates lower than 10% (Fig. 3, Table S1). This suggests that, for species with error rates greater than 10%, obtaining at least 4,500 images would be an important goal for improving model performance. A challenge, however, is that the species with low training sample sizes are generally rarer in nature and/or have a restricted range. This rarity reduces their occurrence in databases such as Bumble Bee Watch because these species are not frequently encountered by volunteers. Some images can be gathered from preserved specimens in collections, but it will also be necessary to mount sampling expeditions specifically aimed at capturing images of these rare species in the field. Our classification model could also make use of images already in hand that have not yet been validated. Bumble Bee Watch, for example, has a backlog of thousands of images that have not been verified by experts, most of which are common species. Passing these images through our model could flag potential high-value images for priority validation by experts. Once validated, the images could be incorporated into subsequent versions of the classification model.

### Maximizing confidence in model predictions

Given the current model, a number of steps can be taken to increase one’s confidence in the model predictions. For example, users can increase accuracy by inputting higher-quality images. Misclassified images are often low-resolution because the bee is small in the original image and therefore not represented by a sufficient number of pixels to capture important features. A bee may also be in poor focus or partially obscured (Fig. 4 A-C). An automated image quality score, based on the number of pixels and focus of the input image, could potentially be provided to users as a screening tool to help them assess their confidence in class predictions.

Users may increase their confidence in a prediction by assessing the results of a series of images of the same individual taken from different points of view. Experts often use an ensemble approach in which they examine multiple images that may each capture different diagnostic features and then assign an identification to all images, even if an identification couldn’t be made based on any single image. The same approach could be taken with our model by assessing the top 3–5 prediction probabilities on a series of photos or frames from a short video clip. We examined a subset of misclassified test images and found that other images of the same individual were often classified correctly.

### Model improvement

Further development of our training data set should enhance generalization and model accuracy, especially by prioritizing images of poorly sampled species with higher error rates. However, exploring new ways of learning and data generation may further enhance the model. For example, metric learning could be used to learn similar features between a pair of images, thus enhancing the discriminative power of deep CNNs^37^. Alternatively, generative adversarial networks^38^ may help improve error rates for poorly sampled species and low accuracy due to class imbalance by generating synthetic image data when new images are difficult to acquire.

Knowledge of the spatial location of an observation may improve model performance as bumble bees vary in appearance across their geographic range. For example, the local phenotype of *B. melanopygus* may more closely resemble *B. bifarius* than its own phenotype from a different portion of its range. Associating learned features with geographic locations may therefore help to improve classification accuracy when observation coordinates are available. Similarly, location data could weight or narrow the prediction field. For example, the individual shown in Fig. 4F was (wrongly) classified as *B. ternarius*, even though it was observed out of the typical range of *B. ternarius*. Removing or downweighting out-of-range predictions would have resulted in the correct prediction, *B. huntii*, which was second most likely.

### BeeMachine web application

We created a web application called BeeMachine to let users identify bumble bees in their own images using our classification model, which can be found at https://beemachine.ai. Users can upload images of bumble bees and receive the top three predictions along with associated probabilities. BeeMachine is in the early stages of development and will be frequently updated to enhance usability and accuracy as well as including more species from other regions of the world in the classification model. BeeMachine can currently be accessed and used on both desktop and mobile browsers but a dedicated mobile application is in development which will allow for a more streamlined user experience in the field, more educational content, and integration into video-based sampling strategies.

## Conclusions

Computer vision will soon play an integral role in bee research. Now common in other fields, classification and object detection models will be used in the lab and deployed on devices in the field to capture data in realtime and over large spatial scales^39^. Further development of this technology and data sets for training models will be critical to our ability to efficiently assess trends in bumble bees and other bee taxa. For example, large scale and ambitious bee monitoring programs, such as proposed by Woodard et al.^40^ could benefit from machine-aided observation and identification technology. Beyond bees, this technology is easily scalable and can be generalized to other taxa. With mounting evidence of a global decline in insect biodiversity^41,42^, we need these AI-based tools to efficiently monitor insect populations. Object detection can be coupled with still or video cameras for automated sampling of insects visiting flowers^43^, captured in traps^44^, or visiting non-lethal camera traps as commonly used in studies of larger wildlife^45^. For example, cameras could be mounted to blue vane or pan traps that have been modified to allow insect visitors to pass through unharmed after triggering an AI-based imaging system. By aiding with hard-to-identify taxa, such as insects, in combination with high throughput systems, applied computer vision can provide a more nuanced picture of global insect biodiversity trends compared to trends in relatively simple measures of biomass e.g.,^46^.

Presently, our model can reliably identify many of the common species of North American bumble bees. Thus, computer vision can already help reduce the workload on overburdened experts, freeing them to focus on more challenging identifications and the science of taxonomy. Continued refinement and taxonomic expansion of machine algorithms, such as ours, will only increase their utility and expand their use to other taxa. But given the fluid nature of taxonomic classification, this tool will remain a work-in-progress, requiring input from a community of taxonomic experts to, among other things, define species. The recent separation of *Bombus bifarius* into two species, *B. vancouverensis* and *B. bifarius*^47^, is but one example of the vital work of taxonomic and genomic experts that will be incorporated into updated versions of our classification model.

## Supplementary Information

Supplementary Information
