# Supplementary material for: Assessing the potential for deep learning and computer vision to identify bumble bee species from images
Source: Sci Rep. 2021 Apr 7;11:7580. doi: 10.1038/s41598-021-87210-1 (PMC8027374; doi:10.1038/s41598-021-87210-1)
Supplement: Supplementary file 1 — Supplementary Information [file 41598_2021_87210_MOESM1_ESM.docx]

Assessing the potential for deep learning and computer vision to identify bumble bee species from images

Brian J. Spiesman^1^* Claudio Gratton^2^ Richard G. Hatfield^3^ William H. Hsu^4^ Sarina Jepsen^3^

Brian McCornack^1^ Krushi Patel^5^ Guanghui Wang^5,6^

1. Department of Entomology, Kansas State University, Manhattan, KS, USA
2. Department of Entomology, University of Wisconsin – Madison, Madison, WI, USA 3 The Xerces Society for Invertebrate Conservation, Portland, OR, USA
3. Department of Computer Science, Kansas State University, Manhattan, KS, USA
4. Department of Electrical Engineering and Computer Science, University of Kansas, Lawrence, KS, USA
5. Department of Computer Science, Ryerson University, Toronto, ON, Canada

*Corresponding Author: email: [bspiesman@ksu.edu](mailto:bspiesman@ksu.edu)

**Supplementary Material**

**Table S1.** Species-level performance on InceptionV3.

| **Species** | **Recall** | **Precision** | **F1-score** | **Error rate (false positive**  **or 1 – precision)** | **Num test images** | **Num training images** |
| --- | --- | --- | --- | --- | --- | --- |
| *Bombus affinis* | 0.969 | 0.977 | 0.973 | 0.023 | 258 | 1032 |
| *B. appositus* | 0.856 | 0.931 | 0.892 | 0.069 | 174 | 696 |
| *B. auricomus* | 0.924 | 0.893 | 0.908 | 0.107 | 343 | 1372 |
| *B. bifarius* | 0.931 | 0.935 | 0.933 | 0.065 | 1003 | 4012 |
| *B. bimaculatus* | 0.925 | 0.902 | 0.913 | 0.098 | 1087 | 4348 |
| *B. borealis* | 0.914 | 0.949 | 0.931 | 0.051 | 406 | 1624 |
| *B. caliginosus* | 0.407 | 0.846 | 0.550 | 0.154 | 27 | 108 |
| *B. centralis* | 0.843 | 0.858 | 0.850 | 0.142 | 344 | 1376 |
| *B. citrinus* | 0.889 | 0.874 | 0.882 | 0.126 | 226 | 904 |
| *B. crotchii* | 0.906 | 0.906 | 0.906 | 0.094 | 64 | 256 |
| *B. cryptarum* | 0.857 | 0.971 | 0.910 | 0.029 | 77 | 308 |
| *B. flavidus* | 0.785 | 0.820 | 0.802 | 0.180 | 186 | 744 |
| *B. fervidus* | 0.883 | 0.887 | 0.885 | 0.113 | 854 | 3416 |
| *B. flavifrons* | 0.879 | 0.854 | 0.867 | 0.146 | 746 | 2984 |
| *B. fraternus* | 0.947 | 0.960 | 0.954 | 0.040 | 76 | 304 |
| *B. frigidus* | 0.654 | 0.739 | 0.694 | 0.261 | 26 | 104 |
| *B. griseocollis* | 0.973 | 0.938 | 0.955 | 0.062 | 1332 | 5328 |
| *B. huntii* | 0.910 | 0.941 | 0.925 | 0.059 | 614 | 2456 |
| *B. impatiens* | 0.940 | 0.963 | 0.951 | 0.037 | 2000 | 8000 |
| *B. insularis* | 0.872 | 0.837 | 0.854 | 0.163 | 235 | 940 |
| *B. melanopygus* | 0.955 | 0.918 | 0.937 | 0.082 | 896 | 3584 |
| *B. mixtus* | 0.895 | 0.899 | 0.897 | 0.101 | 598 | 2392 |
| *B. morrisoni* | 0.824 | 0.857 | 0.840 | 0.143 | 51 | 204 |
| *B. nevadensis* | 0.959 | 0.939 | 0.949 | 0.061 | 370 | 1480 |
| *B. occidentalis* | 0.895 | 0.942 | 0.918 | 0.058 | 181 | 724 |
| *B. pensylvanicus* | 0.951 | 0.956 | 0.953 | 0.044 | 1141 | 4564 |
| *B. perplexus* | 0.928 | 0.914 | 0.921 | 0.086 | 458 | 1832 |
| *B. rufocinctus* | 0.802 | 0.856 | 0.828 | 0.144 | 776 | 3104 |
| *B. sandersoni* | 0.464 | 0.722 | 0.565 | 0.278 | 28 | 112 |
| *B. sitkensis* | 0.632 | 0.727 | 0.676 | 0.273 | 114 | 456 |
| *B. sylvicola* | 0.629 | 0.796 | 0.703 | 0.204 | 62 | 248 |
| *B. ternarius* | 0.956 | 0.937 | 0.946 | 0.063 | 1102 | 4408 |
| *B. terricola* | 0.951 | 0.938 | 0.945 | 0.062 | 367 | 1468 |
| *B. vagans* | 0.850 | 0.825 | 0.837 | 0.175 | 533 | 2132 |
| *B. vandykei* | 0.735 | 0.824 | 0.777 | 0.176 | 83 | 332 |
| *B. vosnesenskii* | 0.981 | 0.941 | 0.961 | 0.059 | 1066 | 4264 |

**Table S2.** Confusion matrix for InceptionV3, standardized by column sums. Values show, for each true species, the percent of test images that were correctly classified (diagonal) and the percent of test images that were misclassified as other species (off-diagonal).

Predicted species


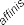


|  | *affinis* |
| --- | --- |
|  | *appositus* |
|  | *auricomus* |
|  | *bifarius* |
|  | *bimaculatus* |
|  | *borealis* |
|  | *caliginosus* |
|  | *centralis* |
|  | *citrinus* |
|  | *crotchii* |
|  | *cryptarum* |
|  | *flavidus* |
|  | *fervidus* |
|  | *flavifrons* |
|  | *fraternus* |
| True species | *frigidus* |
|  | *griseocollis* |
|  | *huntii* |
|  | *impatiens* |
|  | *insularis* |
|  | *melanopygus* |
|  | *mixtus* |
|  | *morrisoni* |
|  | *nevadensis* |
|  | *occidentalis* |
|  | *pensylvanicus* |
|  | *perplexus* |
|  | *rufocinctus* |
|  | *sandersoni* |
|  | *sitkensis* |
|  | *sylvicola* |
|  | *ternarius* |
|  | *terricola* |
|  | *vagans* |
|  | *vandykei* |
|  | *vosnesenskii* |

| 97.7 | 0.0 | 0.0 | 0.0 | 0.0 | 0.0 | 0.0 | 0.0 | 0.9 | 0.0 | 0.0 | 0.0 | 0.2 | 0.0 | 0.0 | 0.0 | 0.1 | 0.0 | 0.0 | 0.0 | 0.0 | 0.0 | 0.0 | 0.0 | 0.0 | 0.0 | 0.0 | 0.0 | 0.0 | 0.0 | 0.0 | 0.0 | 0.0 | 0.5 | 0.0 | 0.0 |
| --- | --- | --- | --- | --- | --- | --- | --- | --- | --- | --- | --- | --- | --- | --- | --- | --- | --- | --- | --- | --- | --- | --- | --- | --- | --- | --- | --- | --- | --- | --- | --- | --- | --- | --- | --- |
| 0.0 | 93.1 | 0.0 | 0.1 | 0.0 | 2.0 | 0.0 | 0.6 | 0.0 | 0.0 | 0.0 | 0.0 | 1.2 | 0.1 | 0.0 | 0.0 | 0.0 | 0.0 | 0.0 | 0.0 | 0.0 | 0.0 | 0.0 | 0.0 | 0.0 | 0.1 | 0.0 | 0.3 | 0.0 | 0.0 | 0.0 | 0.0 | 0.0 | 0.0 | 0.0 | 0.0 |
| 0.0 | 0.0 | 89.3 | 0.0 | 0.0 | 0.0 | 0.0 | 0.0 | 0.0 | 0.0 | 0.0 | 0.0 | 0.0 | 0.0 | 2.7 | 0.0 | 0.0 | 0.0 | 0.0 | 0.0 | 0.0 | 0.0 | 2.0 | 0.8 | 0.0 | 1.1 | 0.0 | 0.4 | 0.0 | 0.0 | 0.0 | 0.1 | 0.5 | 0.0 | 0.0 | 0.1 |
| 0.0 | 0.0 | 0.0 | 93.5 | 0.1 | 0.0 | 0.0 | 0.0 | 0.0 | 0.0 | 0.0 | 1.1 | 0.1 | 0.7 | 0.0 | 0.0 | 0.1 | 1.2 | 0.1 | 1.6 | 1.7 | 0.8 | 0.0 | 0.0 | 0.0 | 0.1 | 0.0 | 0.7 | 0.0 | 0.0 | 0.0 | 1.2 | 0.0 | 0.0 | 5.4 | 0.2 |
| 0.0 | 0.0 | 0.0 | 0.1 | 90.2 | 0.0 | 0.0 | 0.0 | 1.3 | 0.0 | 0.0 | 0.0 | 0.0 | 0.0 | 0.0 | 0.0 | 1.2 | 0.0 | 1.7 | 0.0 | 0.0 | 0.2 | 0.0 | 0.0 | 0.0 | 0.1 | 1.1 | 0.7 | 5.6 | 0.0 | 0.0 | 0.0 | 0.0 | 2.7 | 0.0 | 0.0 |
| 0.4 | 1.3 | 0.6 | 0.0 | 0.0 | 94.9 | 0.0 | 0.0 | 0.0 | 0.0 | 0.0 | 0.6 | 0.6 | 0.0 | 0.0 | 0.0 | 0.0 | 0.0 | 0.0 | 1.6 | 0.2 | 0.0 | 0.0 | 0.0 | 0.0 | 0.7 | 0.4 | 0.3 | 0.0 | 0.0 | 0.0 | 0.3 | 0.5 | 0.0 | 0.0 | 0.1 |
| 0.0 | 0.0 | 0.0 | 0.0 | 0.0 | 0.0 | 84.6 | 0.0 | 0.0 | 0.0 | 0.0 | 0.0 | 0.4 | 0.0 | 0.0 | 0.0 | 0.0 | 0.0 | 0.0 | 0.0 | 0.0 | 0.0 | 0.0 | 0.0 | 0.0 | 0.0 | 0.0 | 0.0 | 0.0 | 0.0 | 0.0 | 0.0 | 0.0 | 0.0 | 0.0 | 1.2 |
| 0.0 | 0.0 | 0.0 | 0.1 | 0.0 | 0.3 | 0.0 | 85.8 | 0.0 | 0.0 | 0.0 | 0.0 | 0.1 | 3.6 | 0.0 | 4.3 | 0.1 | 0.5 | 0.0 | 0.0 | 0.3 | 0.2 | 0.0 | 0.0 | 0.0 | 0.0 | 0.0 | 1.7 | 0.0 | 0.0 | 0.0 | 0.0 | 0.0 | 0.4 | 0.0 | 0.0 |
| 0.0 | 0.0 | 0.0 | 0.0 | 0.3 | 0.0 | 0.0 | 0.0 | 87.4 | 0.0 | 0.0 | 0.6 | 0.1 | 0.0 | 0.0 | 0.0 | 0.1 | 0.0 | 0.1 | 1.2 | 0.0 | 0.2 | 0.0 | 0.0 | 0.0 | 0.2 | 0.4 | 0.4 | 0.0 | 0.0 | 0.0 | 0.0 | 0.0 | 0.9 | 0.0 | 0.0 |
| 0.0 | 0.0 | 0.6 | 0.0 | 0.0 | 0.0 | 0.0 | 0.0 | 0.0 | 90.6 | 0.0 | 0.0 | 0.2 | 0.0 | 0.0 | 0.0 | 0.1 | 0.0 | 0.0 | 0.0 | 0.0 | 0.0 | 0.0 | 0.3 | 0.0 | 0.0 | 0.0 | 0.0 | 0.0 | 0.0 | 0.0 | 0.0 | 0.0 | 0.0 | 0.0 | 0.0 |
| 0.0 | 0.0 | 0.0 | 0.0 | 0.0 | 0.0 | 0.0 | 0.0 | 0.0 | 0.0 | 97.1 | 0.0 | 0.0 | 0.1 | 0.0 | 0.0 | 0.0 | 0.0 | 0.0 | 0.0 | 0.0 | 0.0 | 0.0 | 0.0 | 1.7 | 0.0 | 0.0 | 0.0 | 0.0 | 0.0 | 0.0 | 0.0 | 1.9 | 0.0 | 0.0 | 0.0 |
| 0.0 | 0.0 | 0.0 | 0.2 | 0.2 | 0.0 | 0.0 | 0.0 | 0.9 | 0.0 | 0.0 | 82.0 | 0.8 | 0.0 | 0.0 | 0.0 | 0.0 | 0.0 | 0.0 | 7.3 | 0.1 | 0.0 | 0.0 | 0.0 | 0.0 | 0.0 | 0.0 | 0.7 | 0.0 | 0.0 | 0.0 | 0.0 | 0.0 | 0.0 | 0.0 | 0.3 |
| 0.4 | 4.4 | 0.8 | 0.3 | 0.1 | 0.5 | 7.7 | 0.3 | 0.4 | 3.1 | 0.0 | 1.7 | 88.7 | 0.0 | 0.0 | 0.0 | 0.2 | 0.2 | 0.1 | 2.9 | 0.3 | 0.2 | 2.0 | 1.1 | 1.2 | 1.1 | 0.0 | 1.0 | 0.0 | 0.0 | 0.0 | 0.2 | 0.0 | 0.2 | 0.0 | 2.6 |
| 0.0 | 0.0 | 0.0 | 0.5 | 0.0 | 0.0 | 0.0 | 6.2 | 0.0 | 0.0 | 0.0 | 0.0 | 0.0 | 85.4 | 0.0 | 0.0 | 0.1 | 0.0 | 0.0 | 0.0 | 1.1 | 3.5 | 0.0 | 0.0 | 0.0 | 0.0 | 0.4 | 0.7 | 0.0 | 16.2 | 0.0 | 0.1 | 0.0 | 0.9 | 2.7 | 0.1 |
| 0.0 | 0.0 | 0.6 | 0.0 | 0.0 | 0.0 | 0.0 | 0.0 | 0.0 | 0.0 | 0.0 | 0.0 | 0.0 | 0.0 | 96.0 | 0.0 | 0.1 | 0.0 | 0.0 | 0.0 | 0.0 | 0.0 | 0.0 | 0.3 | 0.0 | 0.0 | 0.0 | 0.0 | 0.0 | 0.0 | 0.0 | 0.0 | 0.0 | 0.0 | 0.0 | 0.0 |
| 0.0 | 0.0 | 0.0 | 0.2 | 0.0 | 0.0 | 0.0 | 0.0 | 0.0 | 0.0 | 0.0 | 0.0 | 0.0 | 0.1 | 0.0 | 73.9 | 0.0 | 0.0 | 0.0 | 0.0 | 0.0 | 0.5 | 0.0 | 0.0 | 0.0 | 0.0 | 0.0 | 0.1 | 5.6 | 0.0 | 0.0 | 0.0 | 0.0 | 0.2 | 0.0 | 0.0 |
| 0.0 | 0.0 | 0.3 | 0.0 | 1.2 | 0.0 | 0.0 | 0.0 | 0.0 | 0.0 | 0.0 | 0.0 | 0.0 | 0.0 | 0.0 | 0.0 | 93.8 | 0.0 | 0.7 | 0.0 | 0.0 | 0.0 | 2.0 | 0.5 | 0.0 | 0.1 | 0.2 | 0.1 | 0.0 | 0.0 | 0.0 | 0.0 | 0.0 | 0.4 | 0.0 | 0.0 |
| 0.0 | 0.0 | 0.0 | 0.1 | 0.0 | 0.3 | 0.0 | 1.8 | 0.0 | 1.6 | 0.0 | 0.0 | 0.1 | 0.3 | 0.0 | 0.0 | 0.0 | 94.1 | 0.1 | 0.0 | 0.9 | 0.0 | 0.0 | 0.0 | 0.0 | 0.0 | 0.0 | 1.0 | 0.0 | 0.0 | 8.2 | 2.0 | 0.0 | 0.0 | 1.4 | 0.0 |
| 0.0 | 0.0 | 0.3 | 0.0 | 5.0 | 0.0 | 0.0 | 0.0 | 1.3 | 0.0 | 0.0 | 0.0 | 0.2 | 0.0 | 0.0 | 0.0 | 2.7 | 0.2 | 96.3 | 0.0 | 0.1 | 0.0 | 0.0 | 0.3 | 0.0 | 0.2 | 0.4 | 0.0 | 0.0 | 0.0 | 0.0 | 0.1 | 0.0 | 2.6 | 0.0 | 0.0 |
| 0.0 | 0.0 | 0.0 | 0.2 | 0.0 | 0.5 | 0.0 | 0.0 | 1.7 | 0.0 | 0.0 | 5.6 | 0.6 | 0.0 | 0.0 | 0.0 | 0.0 | 0.0 | 0.0 | 83.7 | 0.0 | 0.0 | 0.0 | 0.0 | 0.0 | 0.0 | 0.0 | 0.3 | 0.0 | 0.0 | 0.0 | 0.0 | 0.0 | 0.0 | 1.4 | 0.4 |
| 0.0 | 0.0 | 0.0 | 1.2 | 0.0 | 0.0 | 0.0 | 0.9 | 0.0 | 0.0 | 0.0 | 0.6 | 0.0 | 1.3 | 0.0 | 0.0 | 0.0 | 0.3 | 0.1 | 0.4 | 91.8 | 0.3 | 0.0 | 0.0 | 0.0 | 0.0 | 0.0 | 0.4 | 0.0 | 1.0 | 2.0 | 0.3 | 0.0 | 0.0 | 0.0 | 0.0 |
| 0.0 | 0.0 | 0.0 | 0.9 | 0.1 | 0.0 | 0.0 | 0.9 | 0.0 | 0.0 | 0.0 | 0.6 | 0.1 | 2.1 | 0.0 | 4.3 | 0.0 | 0.0 | 0.1 | 0.0 | 1.2 | 89.9 | 0.0 | 0.0 | 0.0 | 0.0 | 0.2 | 0.8 | 0.0 | 10.1 | 0.0 | 0.0 | 0.3 | 0.0 | 0.0 | 0.1 |
| 0.0 | 0.0 | 0.0 | 0.0 | 0.1 | 0.0 | 0.0 | 0.0 | 0.0 | 0.0 | 0.0 | 0.0 | 0.2 | 0.0 | 0.0 | 0.0 | 0.0 | 0.0 | 0.0 | 0.0 | 0.0 | 0.0 | 85.7 | 1.6 | 0.0 | 0.0 | 0.0 | 0.0 | 0.0 | 0.0 | 0.0 | 0.0 | 0.0 | 0.0 | 0.0 | 0.0 |
| 0.0 | 0.0 | 0.6 | 0.1 | 0.0 | 0.0 | 0.0 | 0.0 | 0.0 | 0.0 | 0.0 | 0.0 | 0.1 | 0.0 | 0.0 | 0.0 | 0.1 | 0.0 | 0.0 | 0.0 | 0.0 | 0.0 | 4.1 | 93.9 | 0.0 | 0.1 | 0.4 | 0.4 | 0.0 | 0.0 | 0.0 | 0.0 | 0.0 | 0.0 | 0.0 | 0.1 |
| 0.0 | 0.0 | 0.0 | 0.1 | 0.0 | 0.3 | 7.7 | 0.0 | 0.0 | 0.0 | 1.5 | 1.1 | 0.4 | 0.0 | 0.0 | 0.0 | 0.0 | 0.0 | 0.0 | 0.0 | 0.0 | 0.0 | 0.0 | 0.0 | 94.2 | 0.0 | 0.0 | 0.0 | 0.0 | 0.0 | 0.0 | 0.0 | 1.6 | 0.0 | 1.4 | 0.3 |
| 0.0 | 0.0 | 5.9 | 0.0 | 0.0 | 0.3 | 0.0 | 0.0 | 0.4 | 3.1 | 0.0 | 0.6 | 2.1 | 0.1 | 1.3 | 0.0 | 0.1 | 0.0 | 0.1 | 0.0 | 0.0 | 0.0 | 4.1 | 0.3 | 0.6 | 95.6 | 0.0 | 0.0 | 0.0 | 0.0 | 0.0 | 0.0 | 0.5 | 0.0 | 0.0 | 0.2 |
| 0.4 | 0.0 | 0.0 | 0.0 | 0.6 | 0.0 | 0.0 | 0.3 | 0.0 | 1.6 | 1.5 | 0.0 | 0.1 | 0.0 | 0.0 | 0.0 | 0.0 | 0.0 | 0.1 | 0.0 | 0.0 | 0.3 | 0.0 | 0.3 | 0.0 | 0.0 | 91.4 | 0.4 | 0.0 | 0.0 | 0.0 | 0.2 | 0.0 | 2.2 | 0.0 | 0.0 |
| 1.2 | 0.6 | 0.0 | 1.1 | 0.6 | 0.3 | 0.0 | 2.7 | 4.8 | 0.0 | 0.0 | 3.4 | 2.0 | 0.3 | 0.0 | 4.3 | 1.0 | 0.7 | 0.2 | 1.2 | 0.4 | 1.0 | 0.0 | 0.5 | 0.0 | 0.1 | 0.6 | 85.6 | 5.6 | 0.0 | 6.1 | 1.2 | 0.0 | 4.7 | 1.4 | 0.0 |
| 0.0 | 0.0 | 0.0 | 0.1 | 0.0 | 0.0 | 0.0 | 0.0 | 0.0 | 0.0 | 0.0 | 0.0 | 0.0 | 0.3 | 0.0 | 8.7 | 0.0 | 0.0 | 0.0 | 0.0 | 0.0 | 0.0 | 0.0 | 0.0 | 0.0 | 0.0 | 0.0 | 0.3 | 72.2 | 0.0 | 0.0 | 0.0 | 0.0 | 1.5 | 0.0 | 0.0 |
| 0.0 | 0.0 | 0.0 | 0.0 | 0.0 | 0.0 | 0.0 | 0.0 | 0.0 | 0.0 | 0.0 | 0.0 | 0.0 | 3.8 | 0.0 | 0.0 | 0.0 | 0.0 | 0.0 | 0.0 | 0.1 | 1.7 | 0.0 | 0.0 | 0.0 | 0.0 | 0.0 | 0.1 | 0.0 | 72.7 | 0.0 | 0.0 | 0.3 | 0.0 | 0.0 | 0.0 |
| 0.0 | 0.0 | 0.0 | 0.1 | 0.0 | 0.0 | 0.0 | 0.0 | 0.0 | 0.0 | 0.0 | 0.0 | 0.0 | 0.1 | 0.0 | 0.0 | 0.0 | 0.8 | 0.0 | 0.0 | 0.8 | 0.0 | 0.0 | 0.0 | 0.0 | 0.0 | 0.0 | 0.3 | 0.0 | 0.0 | 79.6 | 0.6 | 0.0 | 0.0 | 0.0 | 0.0 |
| 0.0 | 0.0 | 0.0 | 0.7 | 0.0 | 0.8 | 0.0 | 0.0 | 0.0 | 0.0 | 0.0 | 0.0 | 0.1 | 0.1 | 0.0 | 0.0 | 0.0 | 2.0 | 0.1 | 0.0 | 0.6 | 0.0 | 0.0 | 0.0 | 0.0 | 0.1 | 0.0 | 1.7 | 0.0 | 0.0 | 4.1 | 93.7 | 0.0 | 0.4 | 0.0 | 0.0 |
| 0.0 | 0.0 | 0.8 | 0.0 | 0.1 | 0.0 | 0.0 | 0.0 | 0.0 | 0.0 | 0.0 | 1.1 | 0.1 | 0.0 | 0.0 | 0.0 | 0.1 | 0.0 | 0.1 | 0.0 | 0.0 | 0.2 | 0.0 | 0.0 | 1.7 | 0.4 | 0.0 | 0.0 | 0.0 | 0.0 | 0.0 | 0.0 | 93.8 | 0.0 | 0.0 | 0.0 |
| 0.0 | 0.0 | 0.0 | 0.3 | 1.4 | 0.0 | 0.0 | 0.0 | 0.4 | 0.0 | 0.0 | 0.6 | 0.0 | 0.3 | 0.0 | 4.3 | 0.2 | 0.0 | 0.4 | 0.0 | 0.1 | 0.8 | 0.0 | 0.3 | 0.0 | 0.0 | 4.1 | 1.8 | 11.1 | 0.0 | 0.0 | 0.2 | 0.5 | 82.5 | 0.0 | 0.0 |
| 0.0 | 0.6 | 0.0 | 0.1 | 0.0 | 0.0 | 0.0 | 0.6 | 0.0 | 0.0 | 0.0 | 0.0 | 0.1 | 1.3 | 0.0 | 0.0 | 0.0 | 0.0 | 0.0 | 0.0 | 0.0 | 0.2 | 0.0 | 0.0 | 0.0 | 0.0 | 0.2 | 0.0 | 0.0 | 0.0 | 0.0 | 0.0 | 0.0 | 0.0 | 82.4 | 0.4 |
| 0.0 | 0.0 | 0.3 | 0.0 | 0.0 | 0.0 | 0.0 | 0.0 | 0.4 | 0.0 | 0.0 | 0.6 | 1.2 | 0.0 | 0.0 | 0.0 | 0.0 | 0.0 | 0.0 | 0.0 | 0.2 | 0.0 | 0.0 | 0.0 | 0.6 | 0.1 | 0.0 | 0.0 | 0.0 | 0.0 | 0.0 | 0.0 | 0.0 | 0.0 | 4.1 | 94.1 |
